# Investigation of Path Planning to Reduce Height Errors of Intersection Parts in Wire-Arc Additive Manufacturing

**DOI:** 10.3390/ma14216477

**Published:** 2021-10-28

**Authors:** Gyeong-Hwan Song, Choon-Man Lee, Dong-Hyeon Kim

**Affiliations:** 1 School of Smart Manufacturing Engineering, Changwon National University, 20, Changwondaehak-ro, Uichang-gu, Changwon-si 51140, Gyeongsangnam-do, Korea; pameday71@changwon.ac.kr; 2 Department of Mechanical Engineering, Changwon National University, 20, Changwondaehak-ro, Uichang-gu, Changwon-si 51140, Gyeongsangnam-do, Korea; 3 Mechatronics Research Center, Changwon National University, 20, Changwondaehak-ro, Uichang-gu, Changwon-si 51140, Gyeongsangnam-do, Korea

**Keywords:** additive manufacturing, wire arc additive manufacturing, path planning, intersection part, height error

## Abstract

Additive manufacturing (AM) has the advantages of reducing material usage and geometrical complexity compared to subtractive manufacturing. Wire arc additive manufacturing (WAAM) is an additive manufacturing process that can be used to rapidly manufacture medium-and large-sized products. This study deals with the path-planning strategy in WAAM, which can affect the quality of deposited components. It aims at suggesting effective path planning to reduce the height error of intersection parts. A comparative analysis of the bead width and height at the intersection parts was performed to verify the effectiveness of the proposed path. The initial parameters were determined through single-layer deposition experiments, and multi-layer deposition experiments were performed. The resultant height error in the intersection part was 0.8%, while that in the non-intersection part was absent at the maximum height. Path planning is considered to be an effective method.

## 1. Introduction

Additive manufacturing (AM) has emerged as a technology for reducing part cost by reducing the manufacturing time [1]. Wire arc additive manufacturing (WAAM) is an electrical arc-based direct energy deposition (DED) process that has been established as one of the technologies of AM [2]. Thin-walled and complex geometries have mainly been manufactured using WAAM. The WAAM process was applied to principal technologies, such as the aerospace and automobile industries, owing to its excellent material efficiency. According to Karunakaran et al., WAAM is one of the cheapest techniques among other heat source-based AM [3]. For instance, the WAAM process is less expensive than electron beam-based and laser heat-based processes [4]. The electron beam-based process requires a vacuum device or special vacuum room for producing the deposited components [5]. Laser heat-based processes have low laser efficiency in copper, aluminum, and some nonferrous metals, and WAAM is more suitable for the production of such material [6].

However, WAAM has limitations such as deformation, residual stress, height error, and bead geometrical accuracy. Among these, height errors decrease the quality of production [7]. Therefore, more deposition path studies are required to improve the bead quality and appropriate geometry of components with complex shapes [8]. However, since bead shape defects owing to the arc pre-set occurred in conventional path planning, effective path planning considering these defects is required.

Recently, researchers are paying attention to how deposition bead quality affects the final product. In addition, studies on path planning for bead quality improvement are usually performed in multi-layers. Ding et al. studied the error in bead height according to the bead spacing in single-layer deposition [9]. Li et al. proposed a bead model using overlapped deposition spacing and height [10]. Graf et al. suggested a deposition method to correct the height error in which the experiment was conducted by setting the deposition start point in reverse at each layer [11]. Michel et al. studied the path planning of a feature-based design by developing slicing software [12]. Ma et al. investigated an adaptive path planning method for robotic WAAM of thin-walled structures with varying thicknesses [13]. The deposition with weaving technology was studied. Based on the model of deposition with weaving, an adaptive path planning method for WAAM was proposed. The resulting paths were able to fill thin-walled structures with varying thickness less than twice of the single-bead width. Ding et al. studied a methodology of generating medial axis transform (MAT)-based paths for an arbitrary geometry, either thin-walled or solid structures, with or without internal holes [14]. Moreover, the optimal step-over distance corresponding to the maximum material efficiency was discussed for various geometries. Chen et al. proposed a WAAM system based on the principle of the cold metal process (CMT) and milling machine path conversion of CNC machine tools [15]. The 3D model path was constructed using off-line 3D route simulation software. Zhang et al. studied the path selection of a crack at the interface either perpendicular or parallel to the interface from the viewpoints of microstructure, residual stress, and bi-material system [16]. In the study of Zhang et al., the cause of residual stress and microstructural change was the material properties. Therefore, a study of the path planning is expected as a solution to increase stability by reducing heat accumulation. Szost et al. investigated residual stresses generated and microstructure in the case of Ti samples produced by CLAD and WAAM techniques [17]. Wang et al. proposed intelligent optimization algorithms for solving multi-objective optimization algorithms [18]. Zhang et al. investigated a path-planning strategy to fill a randomly generated polygon with triangular holes and contour paths to reduce residual stress [19]. In addition, because paths are designed for a variety of components, the deposited paths affect the quality of the welding beads and welding products. Ding et al. studied several path planning methods for massive structures [8], while Michel et al. focused on increasing in quality of components deposited using the WAAM process from 2.5D [12]. Kazanas et al. suggested a feature-based design to investigate the production of less material waste [20]. In the path planning design, bead overlap at the intersection was considered that occurring as a factor reducing bead quality. Venturini et al. proposed the optimization of the deposition patterns of T-crossing features [21]. Mehnen et al. proposed a path strategy for shapes such as half-pipes and X cross intersections [22].

The purpose of this study is to suggest effective path planning for intersection parts in WAAM in order to reduce height error at intersections by a continuous and effective deposition. Moreover, the suggested path planning was investigated to increase the internal stability of the deposition product by maintaining the maximum strength with the minimum amount of material by using a grid structure, utilizing the overlapping of beads. Experiments were performed to measure and verify the height error through single-layer and multi-layer depositions. Overlapping beads were used to improve the internal structural stability during the experiments.

## 2. Path Planning for Intersection Part

### 2.1. Problem Statement

**Figure 1:**
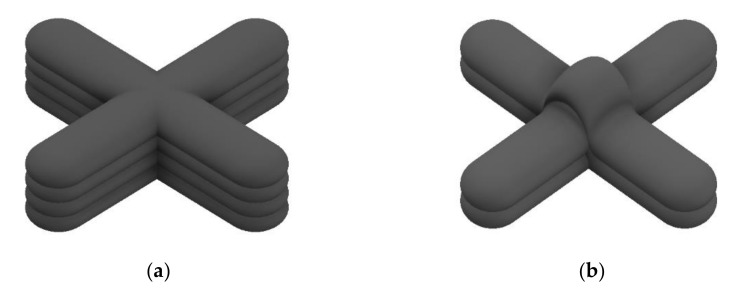
Designs of intersection parts: (**a**) ideal model of deposition products with intersection paths, (**b**) deposit failure that occurs when the deposition process neglects the overlap of the bead by the manufacturing characteristics.

Figure 1 shows an ideal intersection model and a practical problem in the WAAM process. Figure 1a, the ideal model of deposition products with intersecting paths lacks height error, porosity, or manufacturing defects. Figure 1b shows a deposit failure that occurs when the deposition process neglects the overlap of the bead by the manufacturing characteristics. Path planning that neglects the width and height of the deposited bead causes height errors owing to overlapping beads at intersections during multi-layer deposition, which reduces the geometric accuracy.

**Figure 2:**
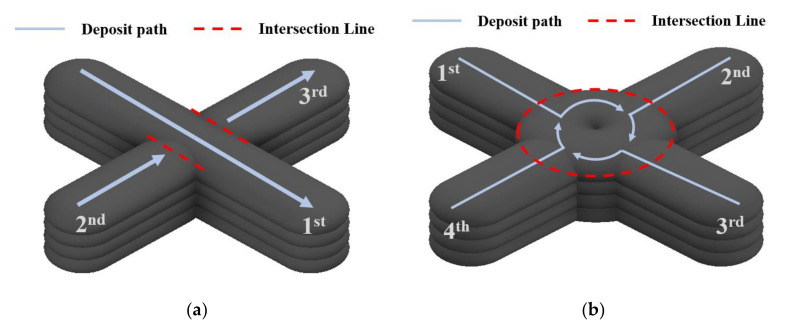
Conventional path planning methods for intersection part: (**a**) the divided path [23], (**b**) the circular path [24].

Figure 2 shows the conventional path planning to reduce the intersection height error. Figure 2a shows the divided path planning of intersections. Although the height error of the divided path planning at the intersection was less than 1 mm, an undercut defect occurred on the contact surface of the bead, so it was not suitable for the path planning of the intersections. However, the limitation of the divided path was that the deposited beads were low precision. In addition, a divided path is unsuitable for path planning to improve internal structural stability. Figure 2b shows the circular path planning to reduce the height error. Circular path planning was studied to reduce the height error by converting the intersection to a circle. However, a height error at the end of the bead due to a discontinuous deposition path was expected, and the path was limited depending on the diameter of the circle or the width of the deposition bead, resulting in a cavity at the center of the intersection.

**Figure 3:**
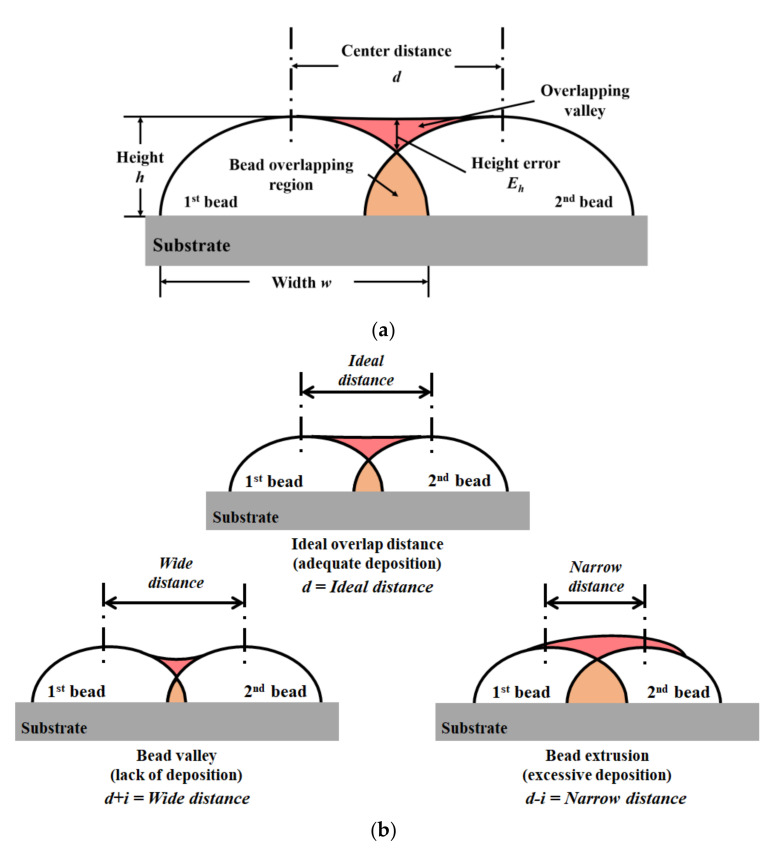
Schematic diagram of the overlapping bead modeling: (**a**) the overlapping model, (**b**) the overlapping model considering height error.

Figure 3 shows the multi-path overlapping of the bead model following the center distance. Figure 3a shows the overlapping bead model, in which the bead has a height error by an interval of the bead. Figure 3b shows the case of the bead overlap model considering the height error. The center distance (d) imposes an effective condition on the height error occurring in the path planning. At the ideal center distance, the height error was almost nonexistent. If the center distance was wide, the small overlapping area caused a height error, and a bead valley was formed. When the center distance was narrow, a height error occurred because of excessive molten metal. Therefore, the ideal d-value was set before the experiments.

### 2.2. Suggested Path Planning

**Figure 4:**
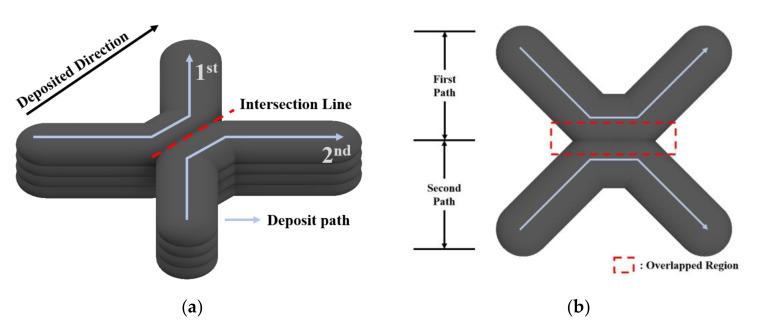
Suggested path planning in this paper. (**a**) a schematic diagram, (**b**) top view.

Effective path planning was proposed to reduce the height error using the bead shape characteristics. The bead overlap model was also utilized in the intersection deposition path to improve the internal structural stability. For bead deposition, the wire feed speed and travel speed affect the bead height and width significantly. Therefore, the interval between the deposition beads was set to reduce the height error of the overlapping part using the characteristics of the overlapping beads. Figure 4 shows the path planning utilizing bead overlap suggested for enhancing bead shape accuracy and internal structural stability. The suggested path planning has the advantage of reducing unnecessary paths, less post-processing, and depositing intersections with minimal height difference using the overlap of beads.

## 3. Materials and Methods

### 3.1. Experimental Setup

**Figure 5:**
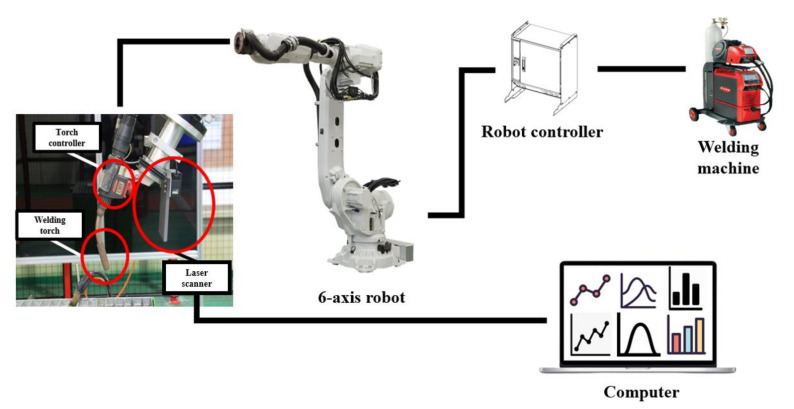
Experimental setup of the WAAM system.

WAAM experiments were performed using the system shown in Figure 5. The 3D line laser scanner (Keyence GmbH., Osaka, Japan) was combined with a torch head to perform the experiments. A computer was used to analyze and collect data on the deposition bead width and height. The 6-axis robot arm (IRB 6700 of ABB Ltd., Zurich, Switzerland) was used to control the motion of the robot with a programmed path and welding process. A welding machine (TPS 500i of Fronius co., Ltd., Pettenbach, Austria) for the metal inert gas welding (MIG) was used for the controlled arc welding process. A laser scanner was used to measure the width and height of the deposited beads. The hardness measurement was conducted by using a rebound durometer (HH-411 of Mitutoyo, Kawasaki, Japan) to obey the experimental conditions of the equipment. Measurements were made 15 times at intervals of 3 mm at a distance of 5 mm from the edge of the specimen.

The experiment was performed using a 304-stainless steel wire (diameter: 1.2 mm). Moreover, the dimensions of substrate were 200 mm × 125 mm × 25 mm. To reduce the heat input of component manufacturing using the cold metal transfer (CMT) mode, a shielding gas was used (99.95% pure argon at a flow rate of 20 ℓ/min). The wire travel speed was set at 5.1 m/min and the travel speed was 5 mm/s. The voltage was set to 120 V depending on the wire feed rate. The deposition parameters used to test the suggested path planning are listed in Table 1.

The 304-stainless steel used in the experiment is one of the representative austenitic stainless steels, with excellent weldability and a wide range of applications such as heat exchangers and transport containers. In addition, it is not oxidized at high temperatures and has excellent corrosion resistance, making it suitable for a wide range of corrosive environments. Cr carbide is formed during prolonged exposure in the temperature range of 425–870 °C, which reduces both corrosion resistance and mechanical strength. However, this experiment was conducted in an environment that does not provide exposure for a long time in the temperature range mentioned above.

### 3.2. Measurements

**Figure 6:**
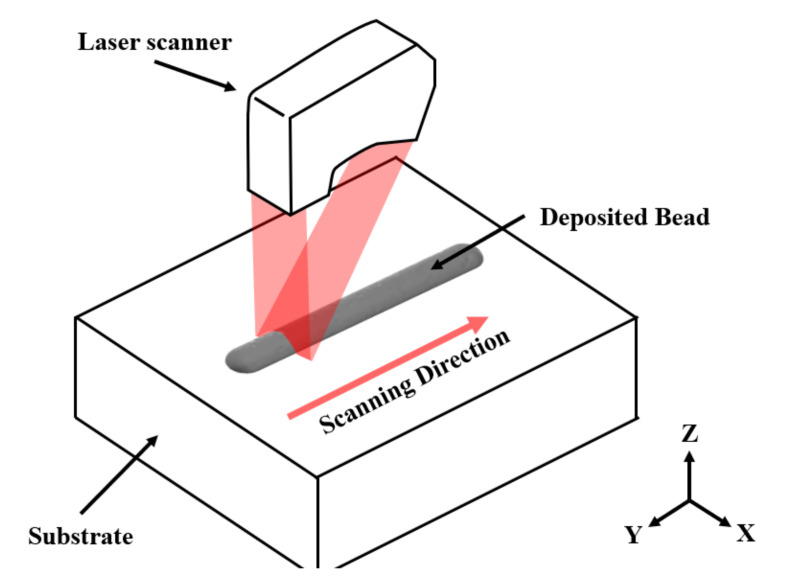
3D triangulation method using a laser scanner. The width and height of the deposited bead were measured.

After the experiment for each layer, the width and height of the deposited bead were measured using a laser scanner, as shown in Figure 6. Measurements were conducted to determine the height error between the intersections and another position on each layer. Measurements were made immediately after deposition on each layer, thereby minimizing changes in height caused by cooling or the surrounding environment. When the height of the deposited bead was checked, the height of the intersection was greater than that of the other position because of the increase in height caused by overlapping beads. The shape of the bead was measured with a laser scanner. In addition, various characteristics, such as internal porosity using SEM, effects of internal stress and microstructure changes via path planning, were checked.

## 4. Results and Discussion

### 4.1. Single-Layer Experiments

**Figure 7:**
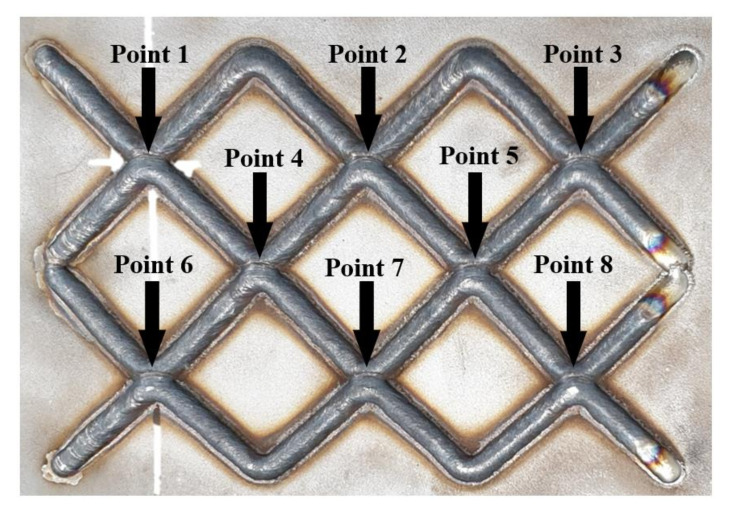
The result of single-layer experiments.

**Figure 8:**
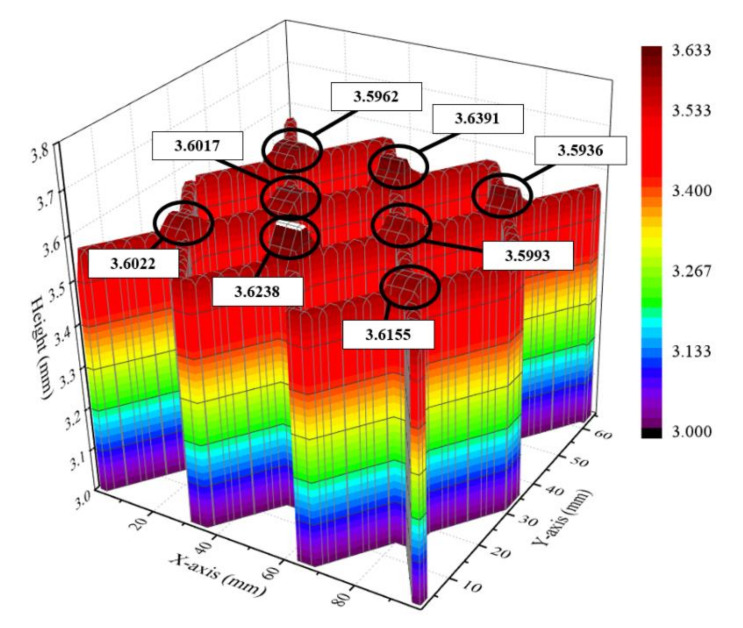
The height value of the single-layer experiment at each point.

Figure 7 shows the results of the single-layer deposition experiment with the intersection path. The path was designed to generate many intersections, while the height of the intersection from points one to eight was measured using a laser scanner. Figure 8 shows the height value of the single-layer experiment at each point. From the results of the single-layer experiment, the average height of the intersection was 3.6089 mm, while the difference between the maximum and minimum heights was 0.455 mm. The error occurred at a magnitude of 1.2% compared to the maximum bead height. Therefore, the single-layer experiment was conducted under a stable deposition.

The multi-layer experiment used the conditions of the single-layer experiment and collected data on the shape of the multi-layer deposition. Then, the height of the intersection was measured and compared to verify that the experimental conditions produced no significant difference even in the multi-layer deposition.

### 4.2. Multi-Layer Experiments

**Figure 9:**
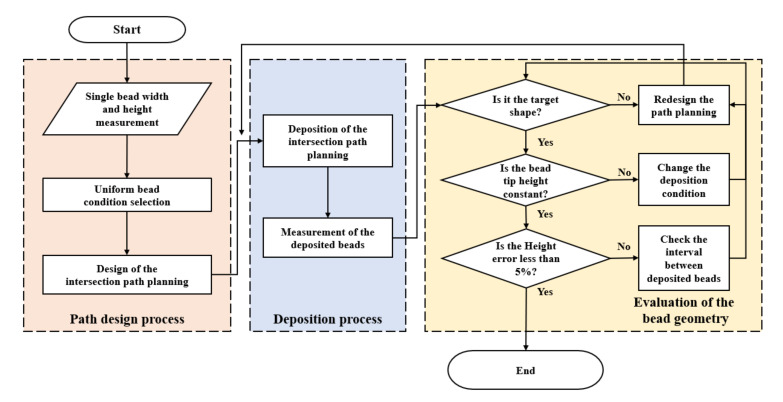
Flowchart of multi-layer intersection deposition in WAAM.

A flow chart was constructed to verify the sequence of the deposition process (Figure 9). A multi-layer deposition experiment using the suggested path planning was designed.

Due to the characteristics of the deposition device, the arc strike occurred by increasing the initial current value for form the bead. Therefore, the height of the front of the bead was manufactured higher than the designed height. To prevent the fusion defects of the molten bead and wire electrode at the end of the deposition path, the wire was not supplied before the end of the deposition. So, the end of the deposition bead was manufactured lower than the designed height as shown in Figure 7. In order to reduce these deposition defects, the deposition direction was reversed in each layer, and the height error of the beads could be reduced.

**Figure 10:**
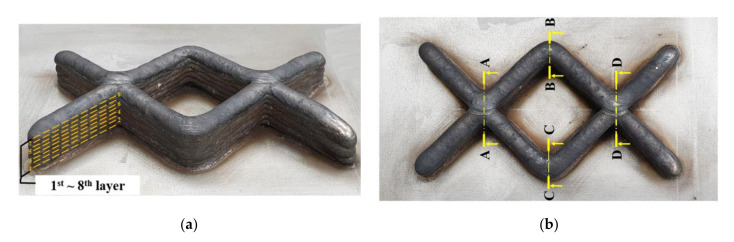
The results of multi-layer experiments: (**a**) the product of multi-layer experiments, (**b**) the height measured sections of intersections.

Figure 10 shows the results of the multi-layer experiment. An eight-layer deposition was stably performed. To verify that the suggested deposition path is free from defects during deposition, height data is collected at four sections, as shown in Figure 10b. Table 2 represents the heights, maximum height difference, and height error rates between the intersections and non-intersections of each layer. The height error rate of each layer was in order to express the height error decrease. The heat was accumulated to each layer during deposition by the heat input and the short cooling time. In the intersections, the heights were 3.5162 mm and 3.7089 mm at the first layer, 6.9075 mm and 6.8437 mm at the second layers, 9.3539 mm and 9.7571 mm at the third layers, 11.8979 mm and 11.7252 mm at the fourth layers, 14.1387 mm and 14.3892 mm at the fifth layers, 16.8256 mm and 16.4218 mm at the sixth layers, 18.8024 mm and 18.8084 mm at the seventh layers, 21.5143 mm and 21.3413 mm at the eighth layers. Moreover, the height errors were 5.2%, 0.9%, 4.2%, 1.4%, 1.7%, 2.4%, 0.03%, and 0.8%, respectively. In the non-intersections, the heights were 3.1573 mm and 3.1573 mm at the first layer, 5.7199 mm and 5.6253 mm at the second layers, 8.0564 mm and 7.9470 mm at the third layers, 10.2608 mm and 10.2608 mm at the fourth layers, 12.8170 mm and 12.7691 mm at the fifth layers, 15.5963 mm and 15.3227 mm at the sixth layers, 17.5123 mm and 17.5016 mm at the seventh layers, 19.7510 mm and 19.7510 mm at the eighth layers. Moreover, the height errors were at 0%, 1.6%, 1.3%, 0%, 0.3%, 1.7%, 0.06%, and 0%, respectively. The height error rate at the intersection was 0.8% at the maximum layer, while the height error in the non-intersection was absent at the maximum layer. This represents negligible height error if surface post-processing is performed to remove irregularities on the top of the deposited product after deposition.

The height errors were measured as shown in Table 2; it was confirmed that the average height error of 0~0.4 mm (0.03~4.2%) occurred. For reliable results, the average values of three experimental iterations under the same conditions were used. The resolution of the laser sensor was four decimal places in mm, and the repeatability was 2 µm. This coincides with other literature in the field [10,23,24], which suggests that height error reductions of 0.05 to 0.30 mm or 0.05~0.5% can occur during WAAM. Table 3 shows the comparison of height error reduction rate with other studies in WAAM.

**Figure 11:**
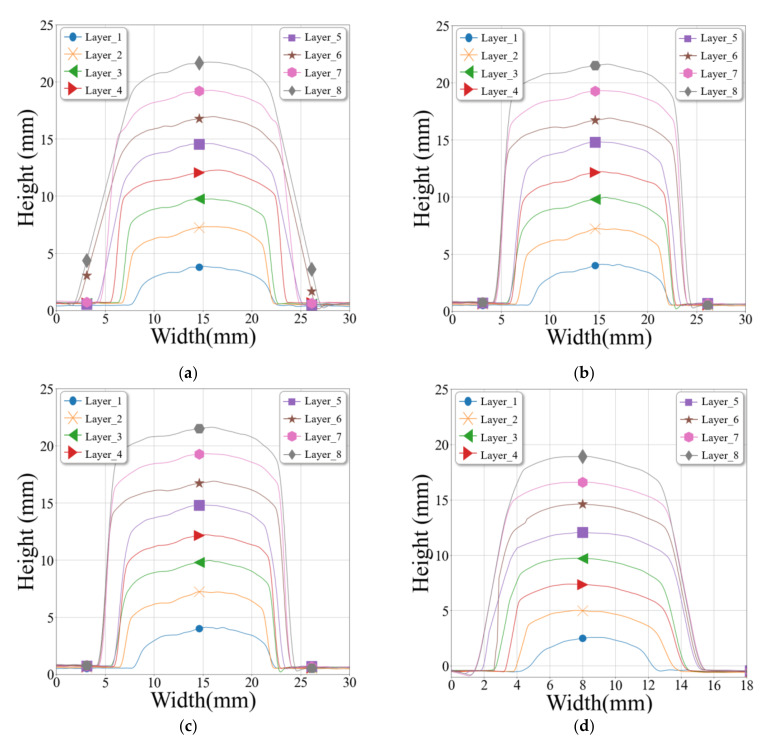
Graph of bead height and width at each layer: (**a**) section view A-A, (**b**) section view D-D, (**c**) section view B-B and (**d**) section view C-C.

Figure 11 shows the height and width of the deposited bead in each layer during the multi-layer experiment. The height of the intersection was higher to the right of the bead at the second path, as shown in Figure 11a,b. The bead heights were checked at the non-intersection part, at the section where the welding direction was shifted, confirming that the height along the central direction was slightly higher than outside, as shown in Figure 11c,d. An additional amount of heat accumulates in each layer because of the arc-based deposition. This heat accumulation influences the process, bead stability, geometrical accuracy, and material properties. Although each layer was assigned the same cooling time, the bead was thickened by heat accumulation. The height difference between the intersection and non-intersection parts was confirmed using the results of the multi-layer experiment.

**Figure 12:**
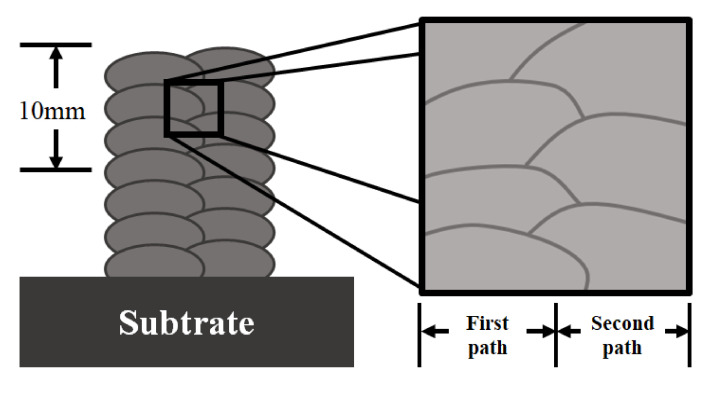
Schematic of measurement at the intersection parts.

**Figure 13:**
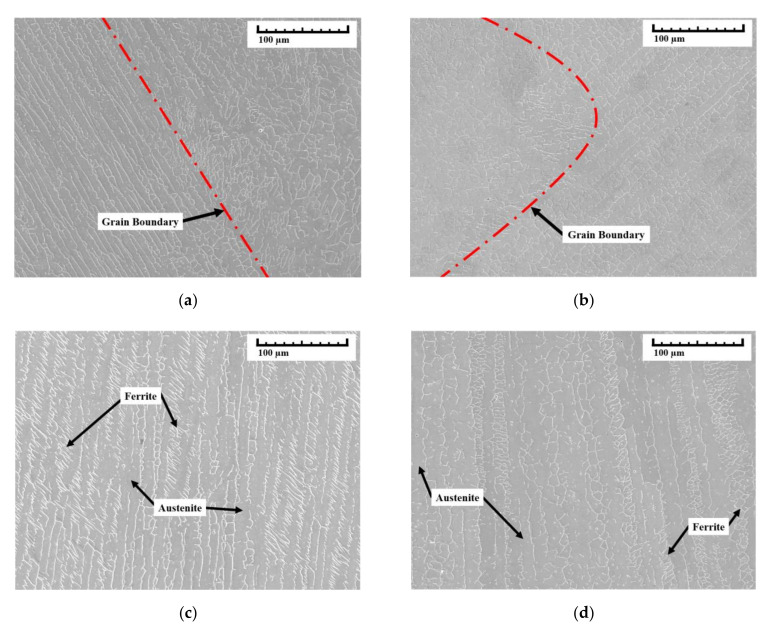
Microstructure of the WAAM-deposited beads studied using SEM: (**a**) section view A-A, (**b**) section view D-D, (**c**) section view B-B, and (**d**) section view C-C.

The multi-layer experiment was performed at the centers of the substrate with eight layers of deposition using the suggested path planning. The microstructure between each layer was analyzed using scanning electron microscopy (SEM). Surface polishing was performed before the SEM measurement, and surface roughness was measured using Portable roughness tester (SJ-210 of Mitutoyo, Kawasaki, Japan). Through three repeated measurements for each specimen, accuracy was improved. The measured average surface roughness of specimens was 0.060~0.083 µm. Figure 12 shows a schematic diagram of component analysis by SEM. The overlapping of beads occurred in sections A-A and D-D, but the shape of the grain boundary was different depending on the measured location. A more detailed analysis of the microstructure of the deposited bead is presented using the SEM image shown in Figure 13. The SEM image of the specimens showed the presence of the ferrite and austenite phases. Figure 13a,b, the SEM images of sections A-A and D-D, show the grain boundaries that appear owing to the overlapping of beads in paths in the components. The cross-sectional views B-B and C-C are shown in Figure 13c,d. The substrate and wire of 304-stainless steel are observed to be perfectly deposited without altering the microstructure of each layer. The SEM image results demonstrate the absence of porosity; however, grain boundaries occurred at the overlapped intersection.

Using specimens whose dimensions were 10 × 10 × 5 mm, the difference in hardness between the intersections and non-intersections was measured. Component analysis was also conducted. The specimens compared height errors, components and were measured.

**Figure 14:**
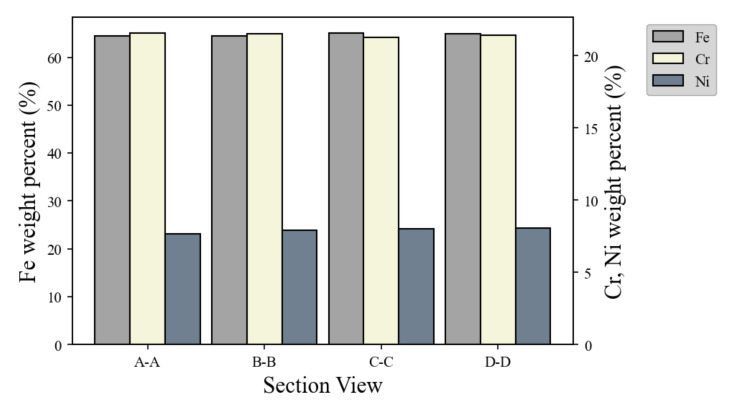
Schematic of measurement at the intersection parts.

Figure 14 shows the results of the analysis of the material components of the specimens at each section. The analysis found that at the intersection, the proportions of Fe, Cr, and Ni were 64.69 wt.%, 21.51 wt.%, and 7.83 wt.%, respectively. At the non-intersection part, the proportions of Fe, Cr, and Ni were 64.8 wt.%, 21.39 wt.%, and 7.96 wt.%, respectively. The intersections had lower mass ratios of Fe and Ni and greater mass ratios of Cr than the non-intersection parts. According to the component analysis, the differences in properties owing to bead overlap between the intersection and non-intersection parts were negligible.

The average Vickers hardness of each specimen was measured. Section A-A showed 136.9 HV, Section B-B showed 114.6 HV, Section C-C showed 123.4 HV, and Section view D-D showed 125.9 HV. According to ASTM A240, the minimum hardness value must be 129. The mean hardness of the intersections, sections A-A and D-D, was 131.4 HV, while the mean value of the non-intersection (sections B-B and C-C) was 119 HV. Although the hardness increased at the intersection where the overlap occurred, it decreased at the non-intersection affected by heat accumulation. The measured Vickers hardness was obtained by using the hardness-tensile strength conversion table (Steel Express Ltd., Wolverhampton, United Kingdom). The tensile strength of the intersection part was about 453.8 MPa and the non-intersection part was 405.3 MPa, showing a difference of 48.5 MPa.

Thus, path planning suggested addressing the problem of arc-based deposition and the decreasing internal structural stability of the WAAM. Its effectiveness was demonstrated in experiments using the WAAM. The suggested path planning was effective to reduce the height error of intersection parts in WAAM.

## 5. Conclusions

The final quality of the deposition beads in the WAAM was considered to be closely linked to path planning. This study suggested reducing the height error and increasing the internal stability using intersection path planning in the WAAM process, thereby necessitating calculations of the bead width and height from the path planning stage. This study was conducted as described below.

(1) The most important step in the WAAM process is path design, which significantly influences the final quality of the product. Conventional intersection or internal path planning do not significantly contribute to height error or improve internal structural stability, owing to their inefficient design. This study suggests path planning utilizing bead overlap to increase the internal structural stability and reduce the height error.
(2) Parameters to reduce height errors in the intersection were selected using the single-layer experiment, and multi-layer experiments were conducted. In the multi-layer experiment, a laser scanner was used to measure the bead width and height of the intersection, non-intersection, and verify the stability. The height error in the intersection part was 0.8%, and it was absent in the non-intersection at the maximum height.
(3) Specimens were produced to investigate the properties and microstructure of the WAAM products, and hardness changes were observed according to the deposition. According to the results of the component analysis and SEM and the hardness measurement, changes in properties owing to deposition were insignificant.

In the suggested path planning design, the bead cooling time and deposition time due to heat accumulation were reduced to increase productivity. The path planning contributes to the productivity improvement of deposited products that can achieve maximum strength with minimal materials by not completely filling the inside of the deposited product using grid structure along with improving the bead stability. Through continuous research, we expect to be able to design the strength of products in the manufacturing process by implementing complex shapes, which is one of the strengths of the AM.

## Figures and Tables

**Table 1:** Process parameters for testing the suggested path planning.

Parameter	Value
Wire diameter	1.2 mm
Current	170 A
Voltage	120 V
Wire feed speed	5.1 m/min
Travel speed	5 mm/s
Contact tip with distance	15 mm
Shield gas/Flow rate	Ar 99.95%/20 ℓ/min
Center distance	2.25 mm

**Table 2:** Comparison of intersections and non-intersections including error rates.

Layer Order	Intersection Part			Non-Intersection Part		
	A (mm)	D (mm)	Error (%)	B (mm)	C (mm)	Error (%)
1st	3.5162	3.7089	5.2	3.1573	3.1573	0
2nd	6.9075	6.8437	0.9	5.7199	5.6253	1.6
3rd	9.3539	9.7571	4.2	8.0564	7.9470	1.3
4th	11.8979	11.7252	1.4	10.2608	10.2608	0
5th	14.1387	14.3892	1.7	12.8170	12.7691	0.3
6th	16.8256	16.4218	2.4	15.5963	15.3227	1.7
7th	18.8024	18.8084	0.03	17.5123	17.5016	0.06
8th	21.5143	21.3413	0.8	19.7510	19.7510	0

**Table 3:** Comparison of height error reduction rate with other studies in WAAM.

Ref. No.	Materials	Height Error Rate (%)	Remarks
-	Steel (SUS-304)	0.03~4.2	Intersection height error
[23]	2319 aluminum alloy	3.4	Suggest path strategy
[25]	Steel (ER70S-6)	~5.77	Prediction bead roughness using machine learning
[26]	Aluminum alloy	75 (reduction compared to before)	Propose adaptive process control scheme (APCS)
[27]	metal-type flux-core wire, Q235 steel	2% (at 50th layer)	Propeller bracket

## Data Availability

The data presented in this study are available on request from the corresponding author.
